# Management of a Unique Subcarinal Bronchogenic Cyst with Robotic-Assisted Thoracic Surgery

**DOI:** 10.7759/cureus.51814

**Published:** 2024-01-07

**Authors:** Sandra Francis, Deepa Treesa Francis, Ishaan Thassu, Mohammad Ahsan Anwaar, Jhanvi Kothari, Rupert Chima, Malavika Jayan

**Affiliations:** 1 Internal Medicine, Windsor University School of Medicine, Basseterre, KNA; 2 College of Medicine, Calcutta National Medical College, Kolkata, IND; 3 Internal Medicine, Combined Military Hospital (CMH) Lahore Medical College and Institute of Dentistry, Lahore, PAK; 4 Internal Medicine, Gujarat Medical Education and Research Society (GMERS) Medical College, Gandhinagar, IND; 5 Internal Medicine, CardioCare Multispecialty Hospital, Abuja, NGA; 6 Internal Medicine, Bangalore Medical College and Research Institute, Bangalore, IND

**Keywords:** computed tomography, subcarinal, posterior mediastinal mass, robotic thoracoscopic surgery, bronchogenic cysts

## Abstract

A bronchogenic cyst (BC), although a rare congenital abnormality, represents the most common cystic lesion in the mediastinum and can present with chest pain and shortness of breath, especially due to compression of adjacent vital structures. The most common diagnostic modalities used are computed tomography (CT) and magnetic resonance imaging (MRI). These cysts may elude even a seasoned clinician unless they become symptomatic. For clinicians attempting to give optimum and prompt management for these cysts, robotic-assisted surgical resection is the recommended treatment of choice. Robotic-assisted thoracic surgery (RATS) offers precision and enhanced visualization, making it a safe and accurate approach for the removal of posterior mediastinal BCs. Our patient is a 65-year-old female who presented with symptomatic posterior mediastinal subcarinal BCs and underwent complete surgical resection with RATS. The diagnosis was confirmed with histopathology. Advancements and the clinical impact of RATS for mediastinal BCs including the Da Vinci robotic surgeries have been demonstrated to be minimally invasive, safe, and feasible especially when in difficult-to-reach areas. RATS has also proven to be advantageous in reducing disease burden and improving patient outcomes.

## Introduction

Bronchogenic cysts (BCs), originating from the ventral foregut, are congenital malformations of the respiratory tract with abnormal budding of the tracheobronchial tree [1]. These cysts can occur ectopically anywhere along the developmental pathway of the foregut [1]. They are most commonly found in the medial or posterior mediastinum with rarer cases seen in the thyroid gland or the heart [2]. These malformations are an iteration of mediastinal lesions that develop into blind pouches filled with fluid and are commonly seen in pediatric populations, with a smaller subset in adults. Sometimes, these cysts can be malignant, and therefore, a complete surgical resection is warranted [2].

Most cysts tend to be asymptomatic and diagnosed incidentally; however, if symptomatic, they present with chest pain, intermittent/persistent cough, dyspnea, dysphagia, and hoarseness. Symptoms occur when these cysts enlarge to compress surrounding structures such as the trachea, esophagus, or recurrent laryngeal nerve, or if a secondary infection arises. When superficial, these cysts develop into sinus tracts but result in an abscess once infected. Rupture of the BCs can lead to hemorrhage and can be life-threatening. Imaging modalities such as computed tomography (CT) or magnetic resonance imaging (MRI) have been demonstrated to be beneficial for diagnostic purposes [1,3]. Definitive diagnosis is achieved by biopsy [1,2]. Biopsy of a BC classically reveals ciliated pseudostratified columnar epithelium and smooth muscle within the wall of the cyst [3,4]. Complete surgical resection is the best treatment. Advancements in surgical techniques and the clinical impact of robot-assisted thoracic surgery (RATS) for mediastinal BCs including the Da Vinci robotic surgeries have been demonstrated to be feasible and minimally invasive. 

We present a case of a BC in the posterior mediastinum. This case highlights the importance of surgery in safely and efficiently managing and demonstrating the promising clinical value of RATS for thoracic mass resection leading to excellent prognosis with no recurrence upon complete resection [1]. Our patient is a 65-year-old woman with chest discomfort and shortness of breath who had no systemic abnormalities. CT scans revealed a massive symptomatic subcarinal BC that needed rapid excision. 

## Case presentation

A 65-year-old female presented with intermittent episodes characterized by a sensation of chest tightness and a perception of something attempting to traverse her chest. She could manage stairs without feeling breathless but occasionally sensed difficulty in food or liquid passage down her chest.

The patient had no significant symptoms or abnormalities in various body systems. She denied fevers, cough, shortness of breath, chest pain, nausea, vomiting, diarrhea, constipation, hematuria, nocturia, discharge, dysuria, back pain, neck pain, joint pain, muscle pain, rash, or pruritus. She also had no changes in vision or eye pain and was alert and oriented without any headaches. All systems reviewed were negative. The patient appeared to be in good health overall with no acute distress; normal eye, head, and neck exams; and no respiratory or neurological issues. 

**Figure 1:**
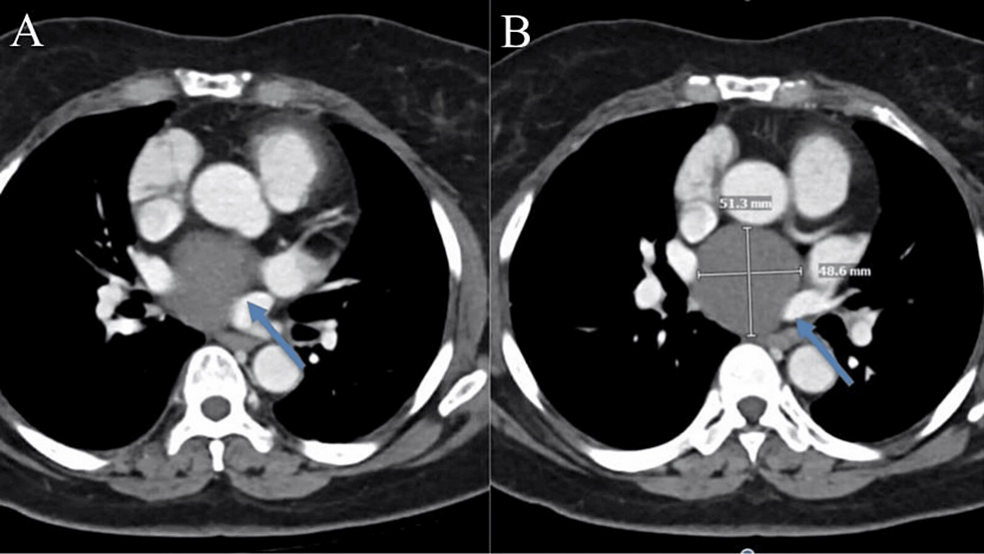
CT scans of the mediastinum and pulmonary hila, showing no lymph node enlargement in the axilla, supraclavicular area, mediastinum, or hilar region. A subcarinal low-density lesion measuring 5.1 × 4.9 cm (A) is observed, which was previously measured as 4.9 × 4.9 cm. No suspicious internal enhancing nodules are detected (B). CT, computed tomography

On performing a CT scan of the chest, a 5 cm complex cyst in a subcarinal area extending next to the heart, as shown in Figure 1, was observed. After consulting with the department of pulmonology, it was revealed to be a large symptomatic subcarinal BC, which required immediate resection. After evaluation with thoracic surgery, resection of the mediastinal mass with robotic surgery was recommended. 

The surgery involved the removal of the cyst from the posterior mediastinum in the subcarinal position. The cyst was identified in the posterior mediastinum in the subcarinal position. The cyst was covered with a thick layer of inflammatory tissue. It ultimately appeared that the cyst was intra-pericardial as the inferior aspect of the cyst could not be determined. The esophagus and bronchus were clearly visualized and were maintained free from the field of dissection at all times. The dissection was made along the superior border of the inferior pulmonary vein. This helped in the identification of a plane between the pulmonary vein and the cyst. At this point, we realized that the cyst was not intra-pericardial. We then proceeded to mobilize the cyst free from the surrounding structures using the bipolar electrocautery at all times. Great care was taken to avoid damage to any of the surrounding structures. The cyst was then crossed to obtain traction to facilitate its dissection and definition of its borders. 

Eventually, the cyst was ruptured. The fluid was completely aspirated. Some of the fluid was sent for culture. We then proceeded to remove the remaining portion of the cyst, which was removed in its entirety leaving no portion of the wall behind. We inspected for hemostasis, which was noted to be adequate. A 19 French Blake drain was then positioned posteriorly in the right chest with portions of it coursing through the surgical bed. The chest was irrigated with large volumes of antibiotic and saline solution and again inspected for hemostasis, which was noted to be adequate. Rib blocks were performed in the standard fashion using bupivacaine. No complications were noted.

The final report of the resected specimen from the department of pathology is as follows: gross: “The specimen is labeled ‘mediastinal cyst’ and demonstrates a segment of pink-tan cystic structure measuring 2.4 x 1.6 x 0.9 cm. The external surface is inked black. The specimen is serially sectioned to reveal a cystic cut surface.” microscopic: “Negative for malignancy.”

## Discussion

BCs are rare lesions that originate from the primitive ventral foregut during gestation. They account for 10-15% of mediastinal tumors and over half of the mediastinal cysts [1]. While they are more commonly found in pediatric patients, they can also be diagnosed in adults, typically during the third and fourth decades of life [1]. The most common locations for BCs in adults are the middle mediastinum and lung parenchyma, with pulmonary cysts causing more symptoms than mediastinal cysts [5].

Symptoms of BCs can include chest pain, shortness of breath, and difficulty swallowing, which are typically due to the compression of adjacent organs. CT and MRI scans are the main diagnostic tools used to identify BCs before surgery. A chest CT scan will show a smooth, round mass in the mediastinum for mediastinal cysts [6].

A histopathologic examination is required to confirm the diagnosis of BCs. The presence of pseudostratified epithelium in the cyst wall is indicative of a BC [7]. In the case presented, the patient was in her seventh decade of life and experiencing unusual symptoms of chest discomfort. A CT scan revealed a large, complex BC (5 cm) in the posterior mediastinum.

Mediastinal BCs are classified into five types based on their location, which include paratracheal, carinal, para esophageal, hilar, and miscellaneous [1]. In this case, the cyst was located in the subcarinal region.

Surgical excision is the accepted treatment for mediastinal BCs, as these patients are at a greater risk of developing symptoms, infections, or rare cases of malignant transformation [6]. The surgical approach has evolved from open thoracotomy to thoracoscopy and now to RATS, which offers the benefit of video-assisted thoracoscopic surgery with improved visualization, reduced hand tremors, and increased precision. The Da Vinci robotic surgical system, with its three-dimensional vision and endo-wrist technology, allows for meticulous dissection in confined spaces, such as the mediastinum [8,9]. While a systematic search of the literature revealed sporadic cases reported on robotic surgical excision of posterior mediastinal BCs, Chen et al., in their study of RATS, concluded that it was safe and feasible for resecting mediastinal masses [10].

This case highlights a symptomatic elderly patient with a large posterior mediastinal BC who underwent successful RATS. It contributes to the limited literature on the various presentations of BCs and confirms that surgery is the mainstay of management. Additionally, it demonstrates the promising clinical value of the robotic surgery system for thoracic mass removal.

## Conclusions

BCs, although more common in the pediatric population, can also be found in adults, generally in the third and fourth decades of life. These cysts can be incidentally discovered when they are asymptomatic. However, when they exert pressure on the surrounding structures, patients may present with symptoms such as chest pain, shortness of breath, and difficulty swallowing, among others. These cysts are mainly diagnosed by CT and MRI scans, and a histopathologic examination to confirm the diagnosis. Advancements and the clinical impact of RATS for mediastinal BCs have been demonstrated to be minimally invasive, safe, and feasible especially when accessing difficult-to-reach areas. 
